# Preoperative Nomogram to Risk Stratify Patients for the Benefit of Trimodality Therapy in Esophageal Adenocarcinoma

**DOI:** 10.1245/s10434-018-6435-4

**Published:** 2018-03-22

**Authors:** Lucas Goense, Peter S. N. van Rossum, Mian Xi, Dipen M. Maru, Brett W. Carter, Gert J. Meijer, Linus Ho, Richard van Hillegersberg, Wayne L. Hofstetter, Steven H. Lin

**Affiliations:** 1 Department of Radiation Oncology, The University of Texas MD Anderson Cancer Center, Houston, TX USA; 2 Department of Radiation Oncology, University Medical Center Utrecht, Utrecht, The Netherlands; 3 Department of Surgery, University Medical Center Utrecht, Utrecht, The Netherlands; 4 Department of Radiation Oncology, Cancer Center, Sun Yat-Sen University, State Key Laboratory of Oncology in South China, Collaborative Innovation Centre for Cancer Medicine, Guangzhou, Guandong China; 5 Department of Pathology, The University of Texas MD Anderson Cancer Center, Houston, TX USA; 6 Department of Diagnostic Radiology, The University of Texas MD Anderson Cancer Center, Houston, TX USA; 7 Department of Gastrointestinal Medical Oncology, The University of Texas MD Anderson Cancer Center, Houston, TX USA; 8 Department of Thoracic and Cardiovascular Surgery, The University of Texas MD Anderson Cancer Center, Houston, TX USA

## Abstract

**Purpose:**

To develop a nomogram that estimates 1-year recurrence-free survival (RFS) after trimodality therapy for esophageal adenocarcinoma and to assess the overall survival (OS) benefit of esophagectomy after chemoradiotherapy (CRT) on the basis of 1-year recurrence risk.

**Methods:**

In total, 568 consecutive patients with potentially resectable esophageal adenocarcinoma who underwent CRT were included for analysis, including 373 patients who underwent esophagectomy after CRT (trimodality therapy), and 195 who did not undergo surgery (bimodality therapy). A nomogram for 1-year RFS was created using a Cox regression model. The upper tertile of the nomogram score was used to stratify patients in low-risk and high-risk groups for 1-year recurrence. The 5-year OS was compared between trimodality and bimodality therapy in low-risk and high-risk patients after propensity score matching, respectively.

**Results:**

Median follow-up for the entire cohort was 62 months. The 5-year OS in the trimodality and bimodality treatment groups was 56.3% (95% confidence interval [CI] 47.9–64.7) and 36.9% (95% CI 31.4–42.4), respectively. The final nomogram for the prediction of 1-year RFS included male gender, poor histologic grade, signet ring cell adenocarcinoma, cN1, cN2-3, and baseline SUV_max_, with accurate calibration and reasonable discrimination (C-statistic: 0.66). Trimodality therapy was associated with improved 5-year OS in low-risk patients (*p *= 0.003), whereas it showed no significant survival benefit in high-risk patients (*p *= 0.302).

**Conclusions:**

The proposed nomogram estimates early recurrence risk. The addition of surgery to CRT provides a clear OS benefit in low-risk patients. The OS benefit of surgery in high-risk patients is less pronounced.

**Electronic supplementary material:**

The online version of this article (10.1245/s10434-018-6435-4) contains supplementary material, which is available to authorized users.

Neoadjuvant chemoradiotherapy (CRT) combined with surgical resection of the esophagus (trimodality therapy) is a generally recommended treatment strategy with curative intent for patients with locally advanced esophageal cancer.^1^,^2^ Definitive CRT (bimodality therapy) is an alternative approach for patients with a poor performance status or inoperable locally advanced esophageal cancer.^3^,^4^ Despite recent improvement in multimodality treatment and perioperative care, esophageal cancer remains a devastating condition for the patient with an estimated 5-year overall survival (OS) rate of 36–47% after trimodality therapy.^5^–^7^

The relatively poor OS even after trimodality therapy is partially attributable to the high incidence (49–85%) of disease recurrence after surgery.^8^ The remaining OS of patients in this setting is generally poor.^9^ To advocate an extensive surgical resection, such as esophagectomy, there should be a fair chance of improving OS combined with an acceptable health-related quality of life.^10^ Despite improvements in (minimally invasive) surgical techniques, esophageal resection can still induce significant treatment-related morbidity and mortality.^11^,^12^ Furthermore, esophagectomy has been associated with a reduction in health-related quality of life up to 3–12 months following surgery.^13^–^15^ As such, in the group of patients who experience early disease recurrence within 1 year of completing their treatment, the benefit of surgery would probably not outweigh its potential side-effects.^13^–^15^ Some suggest that consideration should be given to less invasive treatment strategies in patients who are likely to have early disease recurrence after surgery.^10^ Preoperative identification of these patients may help to guide subsequent treatment decision-making.

Currently, most available studies that assessed prognosis after trimodality therapy rely on the postoperative available pathology results of the resection specimen, limiting their practical use at the time of surgical decision-making.^10^,^16^,^17^ Additionally, no single clinicopathological characteristic in esophageal cancer can yet optimally predict prognosis preoperatively. Therefore, the purpose of the current study was to develop a preoperative risk prediction model for 1-year recurrence-free survival (RFS) after trimodality therapy for esophageal adenocarcinoma—incorporating multiple clinicopathological characteristics and ^18^F-FDG PET/CT features—and assess the OS benefit of subsequent surgery after CRT in patients at low and high risk of early disease recurrence.

## Methods

### Study Population

From a prospectively acquired database, all patients with locally advanced potentially resectable adenocarcinoma of the esophagus (cT1N+ or cT2-4aN_any_) considered eligible for curative resection after initial staging who underwent trimodality therapy or bimodality therapy between January 2006 and February 2016, at the MD Anderson Cancer Center were identified. Patients were excluded if ^18^FDG-PET/CT scanning before and after CRT was not performed or if restaging after CRT discovered distant metastases. Staging was performed in accordance with the 7th edition of the International Union Against Cancer cTNM-classification.^18^ Initial diagnostic work-up included endoscopy with biopsy, endoscopic ultrasound (including fine-needle aspiration if indicated), and ^18^F-FDG PET/CT. The cT-status and cN-status reported in this study were determined before the start of CRT. This study was approved by the institutional review board at MD Anderson Cancer Center and the requirement to obtain informed consent was waived. The data were analysed in May 2017.

### Treatment Protocol

CRT consisted of fluoropyrimidine (intravenous or oral) with either a platinum or a taxane compound with concurrent radiotherapy (45 or 50.4 Gy in fractions of 1.8 Gy) (Table 1). Patients were considered to have received trimodality therapy if esophagectomy was performed within 4 months after completion of CRT. Reasons to refrain from surgery (bimodality therapy) included patient and physician choice (e.g., physician preference for observation) or a decline in performance status secondary to CRT. Surgical treatment consisted of either transhiatal esophagectomy with abdominal lymphadenectomy or Ivor Lewis esophagectomy with abdominal and thoracic lymphadenectomy. The choice of technique was at the discretion of the treating surgeon.

**Table 1:** Patient, tumor, restaging, and treatment-related characteristics of patients treated with trimodality or bimodality therapy

Characteristic	Trimodality therapy (*n* = 373)		Bimodality therapy (*n* = 195)		*p* value	Missing, *n*^d^
Baseline staging	Value	%/SD	Value	%/SD		*n* (%)
Gender					0.229	0
Female	36	9.7%	13	6.7%		
Male	337	90.3%	182	93.3%		
Age (year)^a^	60	± 10	68	±9		0
BMI (kg/m^2^)^a^	25.9	± 5.04	27.9	±6.04		0
ECOG performance status					<0.001	
0	160	42.9%	49	25.1%		
1–2	213	57.1%	146	74.9%		
Weight loss					0.810	
< 10%	294	78.8%	152	77.9%		
≥ 10%	79	21.2%	43	22.1%		
Histologic grade						0
Good/moderate	164	44.0%	96	49.2%	0.232	
Poor	209	56.0%	99	50.8%		
Signet ring cell adenocarcinoma					0.453	0
No	317	85.0%	161	82.6%		
Yes	56	15.0%	34	17.4%		
EUS-based tumor length (cm)					0.087	0
< 4	150	40.2%	93	47.7%		
≥ 4	223	59.8%	102	52.3%		
Nontraversability by EUS					0.751	0
No	310	83.1%	160	82.1%		
Yes	63	16.9%	35	17.9%		
Clinical T status (7th)^b^					0.920	0
IB/II	47	12.6%	24	12.3%		
III/IVa	326	87.4%	171	87.7%		
Clinical N status (7th)^b^					0.111	0
cN0	133	35.7%	80	41.0%		
cN1	138	37.0%	77	39.5%		
cN2-3	102	27.3%	38	19.5%		
Maximum lymph node diameter (cm)^c^					0.676	0
< 1	259	69.4%	139	71.2%		
≥ 1	114	30.6%	56	28.8%		
PET avid nodes at baseline					0.138	0
*m*N0	225	60.3%	130	66.7%		
*m*N+	148	39.7%	65	33.3%		
Celiac lymph node involvement					0.155	0
No	354	94.9%	190	97.4%		
Yes	19	5.1%	5	2.6%		
Induction chemotherapy					0.006	0
No	235	63.0%	145	74.4%		
Yes	138	37.0%	50	25.6%		
Chemotherapy regimen					<0.001	0
Oxaliplatin/5-FU	150	40.2%	42	21.5%		
Docetaxel/5-FU	104	27.9%	81	41.5%		
Docetaxel/capecitabine	81	21.7%	44	22.6%		
Other	38	10.2%	28	14.4%		
Total radiation dose (Gy)					0.192	0
45.0	17	4.6%	14	7.2%		
50.4	356	95.4%	181	92.8%		
Postchemoradiation staging						
Subjective assessment ^18^F-FDG PET					0.001	0
No complete response	251	67.3%	103	52.8%		
Clinical complete response	122	32.7%	92	47.2%		
Postchemoradiation endoscopic biopsy					0.066	10 (1.7%)
No residual cancer	319	86.7%	174	91.6%		
Residual cancer	49	13.3%	16	8.4%		
Days from completion CRT to surgery^a^	60	± 19				0

Data are numbers with percentages in parentheses

*ECOG* Eastern Cooperative Oncology Group, *EUS* endoscopic ultrasonography

^a^Expressed as mean ± SD

^b^Classified according to the 7th edition of the International Union Against Cancer (UICC) tumor-node-metastasis (TNM) classification^18^

^c^Lymph node diameter was measured in the short axis by an experienced radiologist on the axial CT images

^d^Number of missing values for each variable before imputation

### Follow-Up

After treatment patients were routinely monitored at intervals of 3 months in the first year, 6 months during the second and third year, and 12 months until 5 years after treatment or until death. The follow-up assessment consisted of routine blood tests, chest/abdominal CT, endoscopy with biopsies, and/or ^18^F-FDG PET/CT. The main endpoint of this study was 1-year RFS after trimodality therapy and was calculated from the day of surgery to either the date of recurrence or end of follow-up (censored at 12 months in case of > 12 months follow-up). OS was calculated from the end of CRT to either date of death or last follow-up (censored at 5 years in case of > 5-year follow-up).

### Preoperative Predictors

Clinical characteristics were derived from the prospective collected departmental registry. Initial selection of predictors for for 1-year RFS were prespecified based on previous literature to prevent overfitting of the model. Categories were based on previously published cut-off points (Table 1).^10^,^19^–^21^

### Statistical Analysis

Missing data were considered at random and handled using imputation with the iterative Markov chain Monte Carlo method.^22^ Kaplan–Meier curves were used to assess RFS and OS, and differences were evaluated by using the log-rank test. Statistical analysis was performed using SPSS version 24.0 (IBM Corp., Armonk, NY) and R 3.1.2 open-source software (http://www.R-project.org; MatchIt, optmatch, rms, Hmisc, mice, packages). A *p* value < 0.05 was considered statistically significant.

### Model Development

For the development of the model for 1-year RFS only trimodality patients were used. In case of high correlated variables (i.e., Spearman rank correlation coefficient *r *≥ 0.6) the easiest measurable factor was included. The initial multivariable Cox regression model was reduced by using backward stepwise elimination and the Akaike Information Criteria (AIC) was used to compare different models. The discriminative ability of the final model for 1-year RFS was evaluated using Harrell’s C-statistic.^23^ For internal validation, the model was subjected to 200 bootstrap resamples to calculate the optimism of the model, after which the C-statistic was adjusted and a shrinkage factor was calculated to correct the β-coefficients. Calibration of the final model, which reflects the agreement between predicted versus actual (observed) outcomes, was visualized with calibration plots after bias correction. The final model was used to construct a nomogram.

### Propensity Score Matching

The upper tertile of the nomogram score was used to stratify patients in low-risk and high-risk groups for recurrence within 1 year. Propensity score matching was used to balance patient characteristics between the trimodality and bimodality group within the different risk strata. A propensity score was generated using logistic regression, based on all covariates presented in Table 2. Subsequently, the nearest-neighbour matching technique was used to generate matched pairs of cases (1:1) using a caliper width of 0.45.^24^ Kaplan–Meier curves were used to compare OS between trimodality and bimodality for low-risk and high-risk groups, respectively.

**Table 2:** Patient, tumor, re-staging, and treatment-related characteristics of patients at low- and high risk of 1-year disease recurrence according to nomogram after propensity score matching

Characteristics	Propensity score matched low-risk patients					Propensity score matched high-risk patients				
	TMT (*n* = 118)		BMT (*n *= 118)		*p* value	TMT (*n* = 54)		BMT (*n *= 54)		*p* value
	Value	%/SD	Value	%/SD		Value	%/SD	Value	%/SD	
Gender (male)	108	91.5%	109	92.4%	0.811	52	96.3%	52	96.3%	0.497
Age (year)^a^	65	7	67	9	0.153	65	8	66	10	0.400
ECOG performance status (1-2)	71	60.2%	79	66.9%	0.279	41	75.9%	44	81.5%	0.481
Weight loss (≥ 10%)	22	18.6%	24	20.3%	0.742	15	27.8%	14	25.9%	0.828
Histologic grade (Poor)	35	29.7%	36	30.5%	0.887	54	100.0%	54	100%	1.000
Signet ring cell adenocarcinoma (Yes)	7	5.9%	9	7.6%	0.156	16	29.6%	19	35.2%	0.537
EUS-based tumor length (≥ 4 cm)	49	41.5%	52	44.1%	0.693	41	75.9%	41	75.9%	1.000
Nontraversability by EUS (yes)	16	13.6%	22	18.6%	0.288	14	25.9%	10	18.5%	0.355
Clinical T status (III/IVa)^b^	95	80.5%	99	83.9%	0.496	53	98.1%	52	96.3%	0.558
Clinical N status										
(cN1)^b^	37	31.4%	35	29.7%	0.926	31	57.4%	33	61.1%	0.890
(cN2-3)	18	15.3%	17	14.4%		19	35.2%	18	33.3%	
FDG avid nodes at baseline (*m*N +)	31	26.3%	32	27.1%	0.883	30	55.6%	29	53.7%	0.847
Celiac lymph node involvement (Yes)	4	3.4%	1	0.8%	0.175	5	9.3%	4	7.4%	0.728
Baseline SUV_max_ (≥ 7)	64	54.2%	64	54.2%	1.000	53	98.1%	53	98.1%	1.000
Induction chemotherapy (yes)	34	28.8%	33	28.0%	0.885	19	35.2%	16	29.6%	0.537
Postchemoradiation staging										
Assessment ^18^F-FDG PET (cCR)	49	41.5%	55	46.6%	0.431	17	31.5%	21	38.9%	0.420
Endoscopic biopsy (RC)	14	11.9%	11	9.3%	0.526	5	9.3%	5	9.3%	1.000

Data are numbers with percentages in parentheses

*TMT* trimodality therapy, *BMT* bimodality therapy, *ECOG* Eastern Cooperative Oncology Group, *EUS* endoscopic ultrasonography, *SUV* standardized uptake value, *cCR* clinical complete response, *RC* residual cancer

^a^Expressed as mean ± SD

^b^Classified according to the 7th edition of the International Union Against Cancer (UICC) tumor-node-metastasis (TNM) classification^18^

## Results

### Patient and Treatment-Related Characteristics

**Fig. 1:**
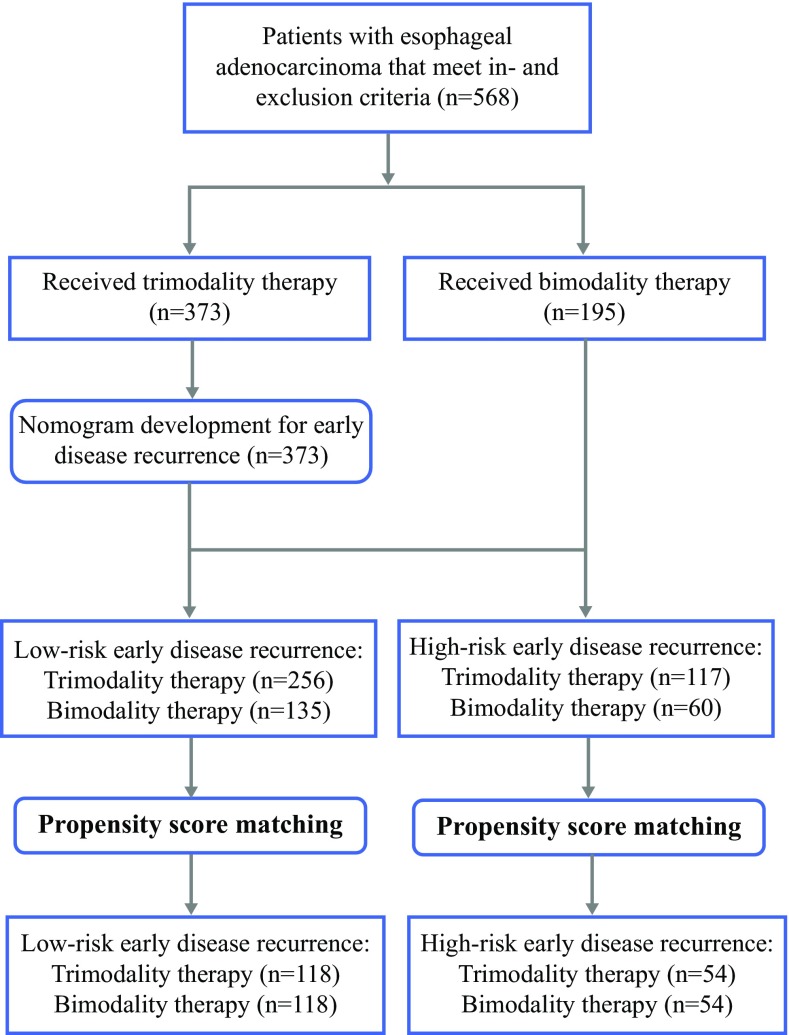
Flow diagram showing study profile

From 568 patients with esophageal adenocarcinoma that met our inclusion and exclusion criteria, 373 underwent trimodality therapy and 195 underwent bimodality therapy (Fig. 1). The distribution of patient and treatment-related characteristics are summarized in Table 1. Of the trimodality patients, 345 (93%) underwent Ivor-Lewis esophagectomy, in 352 (94%) a R0 resection was achieved, and the median number of harvested lymph nodes was 21 (Interquartile range: 15–26). Most common postoperative complications were pulmonary complications (26%), atrial fibrillation (15%), and anastomotic leakage (9%). The median follow-up was 62 months (range 1–130) for the entire cohort. The 5-year OS rate in the trimodality and bimodality treatment groups were 56.3% (95% confidence interval [CI] 47.9–64.7) and 36.9% (95% CI 31.4–42.4), respectively.

### Preoperative Prediction Model for Early Disease Recurrence

Among the 373 trimodality patients, 102 (Kaplan–Meier estimate: 28%) had recurrence within 1 year following esophagectomy, with 91 (89%) having distant metastases. Median OS after documentation of disease recurrence within 1 year after surgery was 9.1 months (95% CI 6.6–11.6). A detailed description of the location and treatment of 1-year disease recurrence is summarized in Supplemental Table 1.

**Fig. 2:**
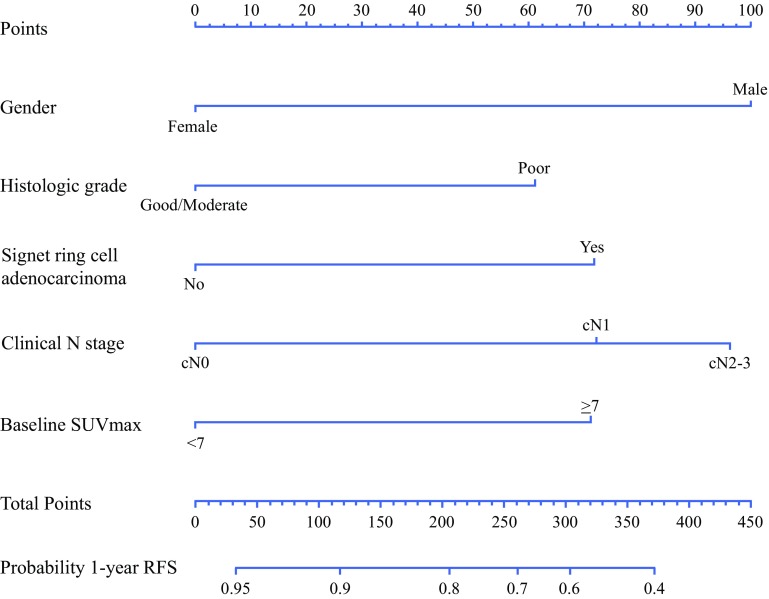
Nomogram for predicting 1-year recurrence-free survival after trimodality therapy

The association of clinical characteristics with 1-year RFS in univariable analysis are presented in supplemental Table 2. After multivariable analysis, male gender (optimism adjusted hazard ratio [aHR] 2.13, 95% CI 0.95–4.77), poor tumor differentiation grade (aHR 1.59, 95% CI 1.07–2.35), signet ring cell adenocarcinoma (cHR 1.72, 95% CI 1.07–2.75), baseline cN1 (aHR 1.72, 95% CI 1.09–2.75), baseline cN2-3 (aHR 2.07, 95% CI 1.27–3.38), and baseline SUV_max_ ≥ 7 (aHR 1.71, 95% CI 1.09–2.69), were independently predictive for 1-year RFS, respectively (Supplemental Table 3). A nomogram based on these variables was constructed (Fig. 2). The discriminative ability of the nomogram was reasonable with an apparent C-statistic of 0.67 and 0.66 after adjustment for optimism. Calibration was accurate, with predictions corresponding closely with the actual observed 1-year RFS probability (Supplemental Fig. 1).

### Risk Stratification of Early Disease Recurrence

Based on the nomogram score patients receiving trimodality treatment were grouped into a low-risk (< 276 nomogram points; number of patients in group = 256) and a high-risk group (≥ 276 nomogram points; number of patients in group = 117) for early disease recurrence, respectively. The corresponding 1-year RFS estimate of the low-risk group (80%) was significantly better than the high-risk group (54%) (log-rank test: *p *< 0.001). After applying the same nomogram score cutoff values to patients in the bimodality group, stratification into low-risk (number of patients in group = 135) and high-risk (number of patients in group = 60) groups allowed significant distinction between 1-year RFS (60 vs. 46%, log-rank test: *p *= 0.049, respectively).

### Survival Comparison Between Trimodality and Bimodality Therapy in Low- and High-Risk Patients

**Fig. 3:**
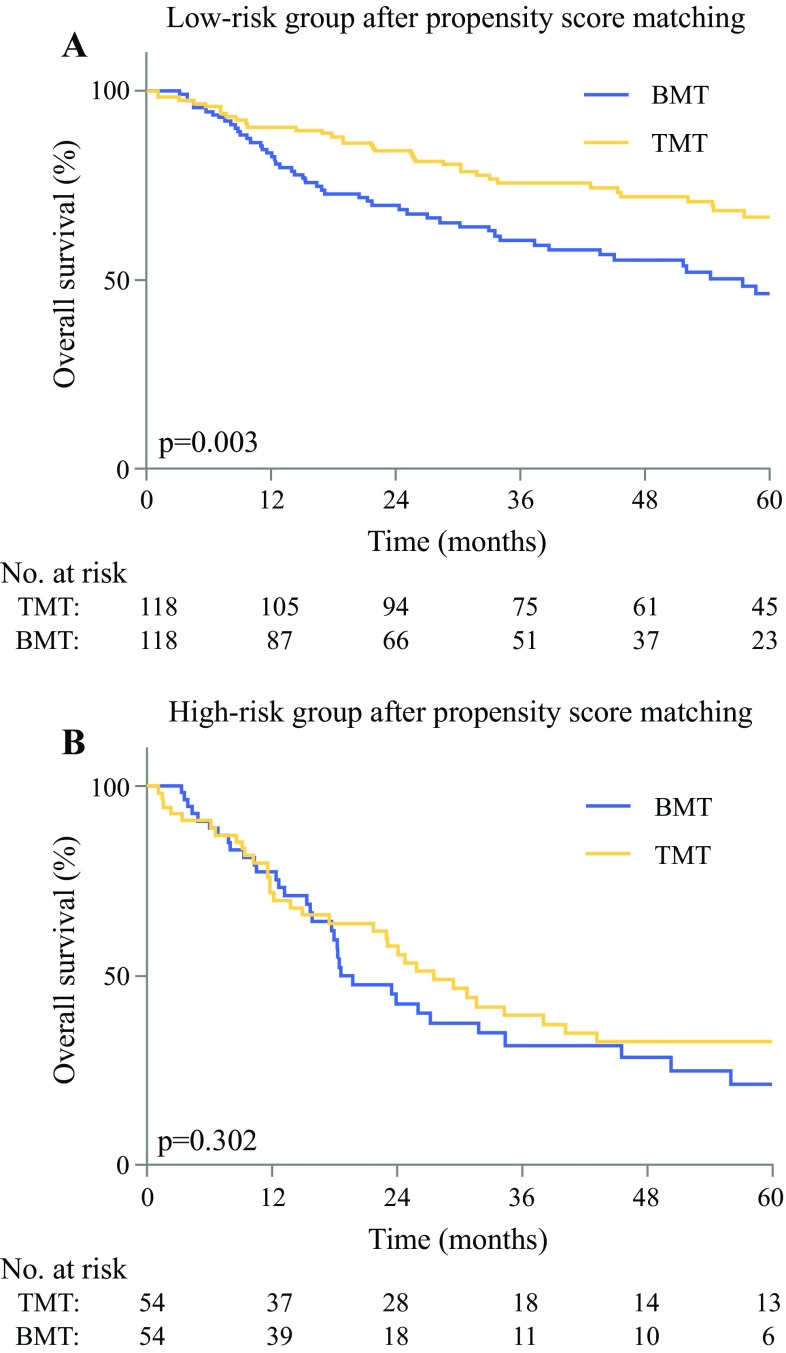
Comparison of overall survival between trimodality and bimodality treatment in the low-risk (**a**) and high-risk (**b**) groups after propensity score matching, respectively

After propensity score matching, balance in patient and tumor characteristics between the stratified trimodality and bimodality groups was achieved (Table 2). In the low-risk group, 5-year OS was significantly better after trimodality therapy compared with bimodality therapy (66 vs. 46%, respectively; log-rank test: *p *= 0.003). In the high-risk patients, 5-year OS difference of trimodality versus bimodality therapy was not statistically significant (32 vs. 21%, respectively; log-rank test: *p *= 0.302, respectively; Fig. 3).

## Discussion

In this study, a preoperative prediction model for early disease recurrence for esophageal cancer patients treated with trimodality therapy was developed. In summary, the proposed nomogram showed accurate calibration and reasonable discrimination (C-statistic: 0.66). Stratification into different risk groups based on the nomogram score allowed significant distinction between 1-year RFS and OS. Treatment with esophagectomy after CRT for patients with a low-risk of early disease recurrence resulted in a substantially higher 5-year OS compared with patients who underwent definitive CRT. Interestingly, the OS benefit of surgery was less apparent (and nonsignificant) in patients with a high-risk of early disease recurrence. Before surgery, by using this easy-to-use scoring system treating physicians could generate individualized predictions on early disease recurrence after surgery. As such, identifying subgroups of patients with different risks of early recurrence may impact shared treatment decision-making and choices of care.

Currently, the NCCN guideline recommends preoperative chemoradiation with subsequent esophagectomy for medically fit patients with locally advanced esophageal cancer.^2^ However, despite multimodality treatment strategies, studies have reported that as many as 29% of the patients experience disease recurrence within 1-year after esophagectomy.^7^ The location of disease recurrence is typically systemic (86–88%) and results in a poor median OS of only 3–9 months.^8^,^25^,^26^ These findings were verified by the current study in which 28% of the patients experienced disease recurrence within 1 year after trimodality therapy (89% systemic), with a median post-recurrence survival of 9 months.

The relatively high incidence of early disease recurrence after trimodality therapy suggests that small distant metastases, which are not detected by currently available staging techniques, may already have occurred at the time of esophagectomy.^20^ Until clinical staging improves significantly, the key point of handling early disease recurrence is to identify high-risk patients and consider alternative treatment strategies. If high-risk patients could be identified accurately, alternative less invasive strategies would be to delay esophagectomy after extensive CRT (with 50.4 Gy) and closely monitor patients for systemic disease. Salvage surgery could then still be an option in high-risk patients who did not develop early systemic recurrence within 1 year.^27^,^28^ Another option would be to avoid chemoradiation due to its considerable morbidity and directly move to esophagectomy.^29^ However, risk stratified treatment pathways in this setting that are most beneficial for patients have yet to be investigated.

The current study identified gender, poor tumor differentiation grade, signet ring cell adenocarcinoma, baseline cN1, cN2-3, and baseline SUV_max_ ≥ 7 as independent prognostic factors for 1-year RFS. These findings are in concordance with previous reports on risk factors for oncologic outcomes (i.e., RFS and OS) after esophagectomy.^10^,^19^–^21^,^29^ By stratifying patients using cutoff values from the proposed nomogram, it was possible to separate patients in low-risk and high-risk groups for 1-year disease recurrence with distinct OS outcomes. For patients with a low-risk profile, the prognosis after trimodality therapy was substantially better compared with patients treated with bimodality therapy.

In the high-risk group, however, patients had a 46% chance of disease recurrence within 1-year after surgery, with no significant OS difference compared to patients treated with bimodality therapy. Because the OS benefit of trimodality therapy in these high-risk patients was considerably less pronounced, an argument could therefore be made to refrain from surgery in these patients. Despite this, most physicians will find it difficult to withhold surgery from a patient with an otherwise resectable tumor based on the predicted outcomes of a nomogram. This is especially true when considering that even some of these high-risk patients are cured after trimodality therapy. Our nomogram should be considered as a first step in the challenging process of patient selection. However, our study at least indicates that a subgroup of patients is likely not served by a multimodality treatment strategy. At best for now, these high-risk patients should be informed about their individual potential for disease recurrence for them to balance the possible risks and benefits of the various treatment strategies.

The discriminative ability of the proposed nomogram may benefit from further refinement with additional predictors in the future. The incorporation of validated risk prediction models for the occurrence and severity of postoperative complications, for example, may further facilitate preoperative decision making.^30^ Furthermore, potential advances that could improve patient selection in the future include blood biomarkers (e.g., circulating tumor DNA) and functional magnetic resonance imaging.^31^–^33^ The latter has shown to have a role in the prediction of pathologic complete response to neoadjuvant CRT.^32^,^33^

Important limitations of this study are that it represents a single-institution analysis, where findings may not be generalizable to other centres. Therefore, external validation of the developed nomogram is warranted to determine generalizability.^34^ Second, although propensity score matching was performed to improve the comparability between the two treatment groups, unknown confounding factors may have influenced our findings. Third, the absence of a statistical significant benefit of esophagectomy in the high-risk patient may be due to a lack of power. As such, the risk-stratified analysis should be validated in a large population. Despite these limitations, the major strengths of this study include that it is the first demonstration of a clinically applicable nomogram for preoperative prediction of 1-year RFS after esophagectomy, providing detailed analyses of handling variables, model building, validation, and calibration according to a standardized template for conducting and reporting of prognostic studies.^35^ This will facilitate validation in other populations and incorporation of other factors to improve this model. Also, the ability of the nomogram to make significant distinction between 1-year RFS in another patient group (i.e., the bimodality group) suggests generalizability of the model.

This study demonstrates a novel nomogram that predicts the preoperative probability of early disease recurrence after trimodality therapy for patients with esophageal cancer. The addition of surgery to CRT provided a clear OS benefit in patients at low risk of early disease recurrence. The OS benefit of surgery in high-risk patients was less pronounced. External validation and improvement of the model with new imaging or biomarkers is desired.

## Electronic supplementary material

Below is the link to the electronic supplementary material.

Supplementary material 1 (DOCX 40 kb)

Supplementary material 2 (TIFF 58 kb)
