# Supplementary material for: Preoperative Nomogram to Risk Stratify Patients for the Benefit of Trimodality Therapy in Esophageal Adenocarcinoma
Source: Ann Surg Oncol. 2018 Mar 22;25(6):1598–607. doi: 10.1245/s10434-018-6435-4 (PMC5928173; doi:10.1245/s10434-018-6435-4)
Supplement: Supplementary file 1 — Supplementary material 1 (DOCX 40 kb) [file 10434_2018_6435_MOESM1_ESM.docx]

**Supplemental Tables:**

| **SUPPLEMENTAL TABLE 1.** Location and treatment of early recurrence within 1-year in 102 patients after esophagectomy with curative intent. | |
| --- | --- |
|  | **n (%)** |
| **Type of recurrence**  Locoregional  Distant  Combined | 11 (11)  71 (70)  20 (19) |
| **Location distant recurrence***  Liver  Lung  Brain  Adrenal gland  Bone  Other | 20 (15)  38 (28)  10 (7)  12 (8)  13 (9)  43 (31) |
| **Number of locations with recurrence**  1  2-3  >3 | 63 (63)  33 (33)  6 (6) |
| **Type of management**  *Treatment focused on tumor reduction*  Chemotherapy  Radiotherapy  Chemoradiation  Surgery  Surgery + chemotherapy  Surgery + radiotherapy  Other    *Best supportive care* | *77 (81)*  52 (52)  8 (8)  9 (9)  2 (2)  1 (1)  4 (4)  1 (1)  *25 (25)* |
| *Total number is more than 102 due to the occurrence of metastasis on multiple locations | |

| **SUPPLEMENTAL TABLE 2.** Recurrence free survival estimates (RFS) subdivided by preoperative prognostic variables in trimodality patients. | | | | | |
| --- | --- | --- | --- | --- | --- |
| **Characteristic** | **n= 373** | **(%)** | **1-year RFS estimate (%)** | | ***p*-value** |
| Gender |  |  |  |  |  |
| Female | 36 | 9.7% | 1yr: | 86% | 0.057 |
| Male | 337 | 90.3% | 1yr: | 70% |  |
| Age (years) |  |  |  |  |  |
| <60 | 165 | 44.2% | 1yr: | 71% | 0.914 |
| ≥60 | 208 | 55.8% | 1yr: | 72% |  |
| Histologic grade |  |  |  |  |  |
| Good/Moderate | 164 | 44.0% | 1yr: | 80% | 0.002 |
| Poor | 209 | 56.0% | 1yr: | 65% |  |
| Signet ring cell adenocarcinoma |  |  |  |  |  |
| No | 317 | 85.0% | 1yr: | 74% | 0.025 |
| Yes | 56 | 15.0% | 1yr: | 60% |  |
| EUS-based tumor length |  |  |  |  |  |
| <4cm | 310 | 83.1% | 1yr: | 76% | 0.080 |
| ≥4cm | 63 | 16.9% | 1yr: | 68% |  |
| Nontraversability by EUS |  |  |  |  |  |
| No | 310 | 83.1% | 1yr: | 73% | 0.140 |
| Yes | 63 | 16.9% | 1yr: | 64% |  |
| Clinical T status (seventh)^a^ |  |  |  |  |  |
| IB/II | 47 | 12.6% | 1yr: | 89% | 0.010 |
| III/IVa | 326 | 87.4% | 1yr: | 69% |  |
| Clinical N status (seventh)^a^ |  |  |  |  |  |
| cN0 | 133 | 35.7% | 1yr: | 83% | <0.001 |
| cN1 | 138 | 37.0% | 1yr: | 69% |  |
| cN2-3 | 102 | 27.3% | 1yr: | 61% |  |
| Maximum Lymph node diameter^b^ |  |  |  |  |  |
| <1cm | 259 | 69.4% | 1yr: | 76% | 0.003 |
| ≥1cm | 114 | 30.6% | 1yr: | 62% |  |
| FDG avid nodes at baseline |  |  |  |  |  |
| *m*N0 | 225 | 60.3% | 1yr: | 76% | 0.023 |
| *m*N+ | 148 | 39.7% | 1yr: | 65% |  |
| Celiac lymph node involvement |  |  |  |  |  |
| No | 354 | 94.9% | 1yr: | 73% | 0.012 |
| Yes | 19 | 5.1% | 1yr: | 53% |  |
| Postchemoradiation endoscopic biopsy |  |  |  |  | 0.063 |
| No residual cancer | 323 | 86.6% | 1yr: | 73% |  |
| Residual cancer | 50 | 13.4% | 1yr: | 61% |  |
| Subjective assessment ^18^F-FDG PET |  |  |  |  |  |
| No complete response | 251 | 67.3% | 1yr: | 69% | 0.071 |
| Clinical complete response | 122 | 32.7% | 1yr: | 77% |  |
| Baseline SUV_max_ |  |  |  |  |  |
| <7 | 117 | 31.4% | 1yr: | 83% | 0.003 |
| ≥7 | 256 | 68.6% | 1yr: | 67% |  |
| Data are numbers, with percentages in parentheses; ^a^Classified according to the 7th edition of the International Union Against Cancer (UICC) tumor-node-metastasis (TNM) classification^18^; ^b:^Lymph node diameter was measured in the short axis by an experienced radiologists on the axial CT images; EUS: endoscopic ultrasonography; SUV: standardized uptake value. | | | | | |

| **SUPPLEMENTAL TABLE 3.** Results of final Cox Proportional Hazards regression model for 1-year recurrence free survival after trimodality therapy*. | | | |
| --- | --- | --- | --- |
| **Characteristic** | **Hazard-Ratio**  (95% CI) | **Adjusted Hazard-Ratio**  (95% CI) | **P value** |
| Gender  (Male vs. Female) | 2.34 (0.94-5.79) | 2.13 (0.95-4.77) | 0.067 |
| Histologic grade  (Poor vs. Good/Moderate) | 1.68 (1.08-2.62) | 1.59 (1.07-2.35) | 0.022 |
| Signet ring cell adenocarcinoma  (yes vs. no) | 1.84 (1.08-3.13) | 1.72 (1.07-2.75) | 0.025 |
| Clinical nodal status  (N1 vs N0 )  (N2 vs N0 ) | 1.85 (1.10-3.12) 2.27 (1.31-3.93) | 1.72 (1.09-2.75) 2.07 (1.27-3.38) | 0.021 0.003 |
| Baseline SUV_max_  (≥7 vs. <7) | 1.83 (1.10-3.04) | 1.71 (1.09-2.69) | 0.019 |
| Shrinkage factor: 0.89. (after 200 bootstrapping resamples);  Initial Harrell’s C statistic: 0.67  Adjusted Harrell’s C statistic: 0.66 (after 200 bootstrapping resamples).  Baseline cumulative survival proportion at 12 months: 0.75.  *Model selection based on Akaike information criterion | | | |

**Supplemental Figure 1:** Calibration plot showing the nomogram predicted versus the observed probability of recurrence free survival at 1-year after esophagectomy. The grey diagonal line represents the optimal line in case of complete concordance between predicted and observed recurrence free survival**.**
