# Supplementary figures and images for: Preoperative Nomogram to Risk Stratify Patients for the Benefit of Trimodality Therapy in Esophageal Adenocarcinoma
Source: Ann Surg Oncol. 2018 Mar 22;25(6):1598–607. doi: 10.1245/s10434-018-6435-4 (PMC5928173; doi:10.1245/s10434-018-6435-4)

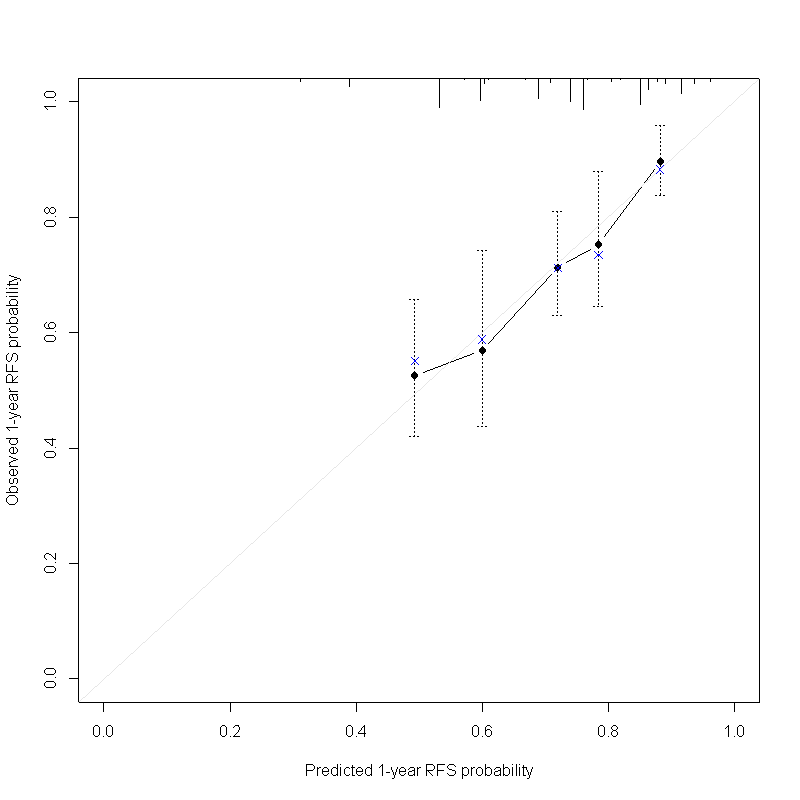

Supplement: Supplementary file 2 — Supplementary material 2 (TIFF 58 kb) [file 10434_2018_6435_MOESM2_ESM.tif]
